# Mentorship in a changing dermatology landscape: new challenges, opportunities, and implications for women

**DOI:** 10.1097/JW9.0000000000000287

**Published:** 2026-08-14

**Authors:** Simona A. Alomary, Nicole J. Baker, Grace Ugwueke, Nada Elbuluk, Temitayo Ogunleye, Susan C. Taylor

**Affiliations:** a Rutgers New Jersey Medical School, Newark, New Jersey; b Department of Dermatology at the Perelman School of Medicine, University of Pennsylvania, Philadelphia, Pennsylvania; c Sidney Kimmel Medical College at Thomas Jefferson University, Philadelphia, Pennsylvania; d Meharry Medical College, Nashville, Tennessee; e Department of Dermatology, University of Southern California Keck School of Medicine, Los Angeles, California

**Keywords:** Career development, medical students, mentorship, mid-career, residents, training

**What is known about this subject in regard to women and their families?**

- Women increasingly pursue dermatology but remain underrepresented in senior and leadership roles.
- Mentorship has long been known to improve career prospects, confidence, and professional development for women in dermatology.
- Access to effective mentorship is uneven across institutions and can be challenging to acquire, which can disproportionately disadvantage women, especially those from underrepresented or less-resourced backgrounds.

**What is new from this article as messages for women and their families?**

- Recent structural changes to the dermatology application process and medical education have made mentorship more valuable than ever for women navigating dermatology.
- As these changes have made the match process more subjective in nature, mentorship functions as a key resource for advocacy, opportunity, and strategy to help guide women toward a more successful match.
- There are no clear guidelines on the impact of these structural changes, but mentors and mentees must both be made aware of these changes so that they can pivot around them.

## Introduction

Women in dermatology often face the challenge of navigating a competitive specialty with limited formal guidance. Students or trainees are commonly responsible for independently seeking guidance and mentorship, as mentorship can play a critical role in both professional and personal development. While mentorship is essential for all medical students and trainees pursuing dermatology, the vast majority of trainees (87.6-98.5%) report mentorship as having a positive impact on their career development.^1–4^ Mentorship may be particularly important for women, who now represent a growing proportion of trainees yet remain underrepresented in leadership positions within the field.^1,2^

Mentorship is generally defined as a longitudinal relationship between an individual with more experience in a particular field or special interest (mentor) and a less experienced individual (mentee). Through a mentorship relationship, the mentor typically offers individualized guidance and support geared toward helping the mentee succeed. This differs from academic advising, which is typically task-oriented and focused on discrete outcomes such as improvement in a course. In contrast, mentorship emphasizes longitudinal growth, formation of a professional identity, and career development. Mentorship may take several forms, including traditional one-on-one longitudinal relationships, group or team-based mentoring, and peer or near-peer mentoring (mentoring between more senior trainees and those at earlier stages of training, for example). More recently, models such as reverse mentoring, in which junior individuals provide insight to more senior colleagues, and micromentoring, consisting of brief targeted interactions focused on specific goals, have also emerged. These models each serve a distinct purpose, from providing longitudinal, individualized guidance to offering more flexible, targeted support and exposure to diverse perspectives.

The role of a mentor includes, but is not limited to, offering insights that prevent career missteps, guidance on career paths, assistance in navigating professional and personal challenges, as well as general support and encouragement. In dermatology, mentorship has been shown to improve satisfaction during training and often inspires mentees to become mentors themselves.^3,4^ While the importance of mentorship has long been recognized, mentorship is variably structured and inconsistently available across institutions.

Mentors can be either appointed or acquired. Both appointed and acquired mentors are useful, but survey data suggest that acquired mentors may be more impactful: 98.5% of respondents rated spontaneous (acquired) mentors as helpful compared with 87.6% for appointed mentors, and they were more likely to provide guidance in workplace politics (70.9 vs 65.5%) and career advancement (86.8 vs 79.7%).^4^ Importantly, having multiple mentors across various domains can provide broader perspectives and is strongly encouraged.^5^ Notably, mentorship remains valuable throughout all stages of a physician’s career. Before and during medical school, mentorship helps individuals assess whether medicine and dermatology are appropriate career paths and can provide early exposure to specialty-specific experiences. During residency, mentorship supports further development of clinical skills, research endeavors, and career decision-making.^6,7^ For early-career dermatologists, junior faculty, and even mid-to-late career dermatologists, mentorship continues to be critical for learning how to establish a professional identity, navigating work promotion, and promoting equity for women and underrepresented groups in dermatology.^8^

Aside from direct mentor–mentee relationships, broader structural and demographic changes within medical education and dermatology have increased the importance of mentorship in the field, particularly for women. Although women represent a growing proportion of medical students (55.1% of matriculants in the 2024 to 2025 academic year) and over 64% of dermatology residents, they remain underrepresented in academic leadership roles and senior academic positions within dermatology.^1,2^ Women hold only 23% of department chair positions and 48% of residency program director roles.^9^ As dermatology continues to become more competitive, unequal access to effective mentorship risks worsening these existing gender disparities. As such, institutional policies that provide protected time, formal recognition, and academic credit for mentorship may be particularly important to support women mentors, who are often disproportionately relied upon yet remain underrepresented in leadership roles.^1,2,9^ This work aimed to understand how recent structural changes in medical education and the dermatology application process could potentially reshape the role of mentorship for women in dermatology.

## Changes in the dermatology application landscape

### The United States Medical Licensing Examination

The transition to a pass/fail United States Medical Licensing Examination Step 1 scoring system in January 2022 has significantly reshaped the dermatology residency application landscape, shifting the evaluation of candidates’ academic ability toward Step 2CK performance, research productivity, and clinical clerkship grades.^10^ Concurrently, many medical schools have adopted pass/fail grading systems for academic coursework and eliminated class rank, removing metrics that previously played an important role in interview selection and determining applicant ranking.^11^ Overall, these changes have promoted increased reliance on more subjective components of the application, including third-year clinical evaluations by faculty, letters of recommendation, and personal statements.^12^ These shifts have increased uncertainty in how applicants can be evaluated, increasing the importance of mentorship in helping students interpret readiness.

### Signaling

Preference signaling, with 3 gold and 25 silver signals, has become a prominent feature of the dermatology residency application process. With 3 gold signals and 25 silver signals, applicants are able to communicate varying levels of interest to residency programs. In turn, programs are able to prioritize applicants who have expressed genuine interest and make more deliberate decisions during the review process. Despite its intent to improve alignment between applicants and programs, preference signaling may inadvertently disadvantage certain applicants, particularly those without access to robust mentorship or institutional guidance.^13^ For example, an applicant may choose not to signal a certain program based on inaccurate self-assessment of competitiveness or lack of guidance on what programs may best align with their future career goals. In addition, geographic prioritization within signaling frameworks may further disadvantage applicants who are open to relocating, lack established regional connections, or train at medical schools without a home dermatology program. These settings often provide fewer opportunities for early exposure, faculty advocacy, and informed guidance on signaling strategies. As a result, mentorship can be critical for applicants when deciding how to use signals intentionally and effectively.

### Letters of recommendation

Following recent changes to the residency application process, 67.6% of program directors across all specialties anticipate that letters of recommendation will assume greater importance in residency selection.^12^ Overall, longitudinal mentorship can lead to effective letters of recommendation for mentees because these letters are written based on personal knowledge of the candidate. For applicants seeking training at academic institutions, letters from academic dermatologists may be particularly helpful, as they provide assessment within an academic clinical context. Such letters may be weighted more heavily than those from nonacademic dermatologists. This may advantage applicants from well-known institutions who may possess established professional networks and greater access to prominent letter writers.^13^ Conversely, applicants from less-resourced institutions or without a home dermatology program may face challenges in securing comparable letters of recommendation, despite similar clinical ability or academic merit. In this context, mentorship can help bridge this gap by connecting trainees with faculty advocates and fostering relationships that may lead to more meaningful letters of recommendation.

### A dedicated research year

A dedicated research year between the third and fourth years of medical school has become increasingly common among dermatology applicants. An analysis of the 2019 and 2020 residency match cycles found that 31.3% of applicants pursued a gap year, with 78.7% aiming to do so to strengthen their application.^14^ However, research indicates that gap years do not consistently improve overall match rates, although they may increase competitiveness for applicants pursuing top-tier programs.^14^ A dedicated research year often requires substantial financial flexibility, as not all are compensated or are inadequately compensated, making this pathway less accessible to applicants with limited financial resources.^15,16^ Mentorship plays a critical role in navigating these barriers, as mentors can help applicants assess whether a research year is necessary, identify funded programs and scholarship opportunities, and offer guidance on how to make the most of the research year experience. Furthermore, strategies to improve equitable access to research years should include the expansion of funded research year positions, transparent application processes, and broader dissemination of resources and opportunities to mitigate financial barriers, such as scholarships and grants.

## Implications for the mentor–mentee relationship

As a result of these recent structural changes, there is more demand for mentors as mentees seek guidance and support throughout the application process. A cross-sectional survey by Vasquez et al.^17^ identified that mentorship and pipeline enrichment programs are essential to being competitive when applying to dermatology residency programs. In addition, research output has also become critically important for a successful match.^18,19^ Mentors can facilitate knowledge of and provide support for participation in research experiences, which significantly help students strengthen their applications. Many programs have been put in place to connect mentors and mentees, including mentorship opportunities established by the American Academy of Dermatology, the Skin of Color Society, as well as other professional dermatology associations or societies. While mentors may feel pressure to take on more mentees, awareness of the formal opportunities that exist in the dermatology community can help distribute responsibilities and provide support to applicants. The development of a standardized mentorship curriculum, including defined expectations, goal-setting frameworks, and regularly scheduled feedback sessions, may help reduce variability in mentorship quality and experiences across institutions.

### Self-reflection and self-evaluation

Effective mentorship requires active participation from the mentee rather than reliance on passive guidance. Furthermore, current mentorship literature emphasizes the importance of mentees engaging in self-reflection to clarify their professional goals, identify personal strengths, and recognize areas for growth before initiating a mentoring relationship.^20^ Preparation before and during formal mentorship allows the mentee to seek mentors whose expertise aligns with their specific needs and facilitates more focused, goal-directed interactions. Entering mentorship relationships with this level of intentionality also promotes clearer communication and efficient use of time.

### Proactive behaviors

In addition to self-reflection and evaluation, active engagement by the mentee throughout the mentoring relationship is essential. Studies consistently highlight practical strategies that strengthen mentor–mentee interactions, including setting clear agendas for meetings, providing regular progress updates, and maintaining honest communication regarding goals and needs.^21–23^ These practices promote alignment of expectations, foster accountability, and enable mentors to offer more targeted and timely guidance. By approaching mentorship as a bidirectional and collaborative process, mentees can maximize the effectiveness and sustainability of relationships with mentors.

### Breadth of mentorship

Relying on a single mentor may be limiting, as professional goals and needs continue to evolve throughout time and an individual may have different areas of interest and challenges. Cultivating a network of diverse mentors offers flexibility to mentees, mitigates situations in which one mentor–mentee relationship is not an ideal fit, provides unique perspectives, and allows different mentors to address different aspects of professional development.^20,24^ Importantly, effective mentorship does not require racial, ethnic, or sex concordance between mentors and mentees; rather, shared values, professional interests and goals, aligned expectations, and cultural awareness are key determinants of mentorship quality.^25^

### Formats for mentorship

Traditionally, mentorship in dermatology was largely based on local institutional relationships, relying heavily on in-person interactions within a single institution or geographic region. This limits mentorship opportunities for individuals without home dermatology programs or strong local representation. More recently, advances in technology and the use of virtual communication tools have facilitated successful mentor–mentee relationships across institutions and regions.^26^ The coronavirus disease 2019 pandemic further necessitated the adoption of virtual mentorship, normalizing remote engagement and supporting hybrid mentorship models that combine in-person and online interactions.^27^ These flexible formats have broadened access to mentorship opportunities and allowed mentoring relationships to extend beyond traditional institutional and geographic boundaries. Effective virtual mentorship must be supported by structured communication, establishing clearly defined expectations, and intentional goal-setting to maintain engagement and accountability across geographically distributed mentor–mentee relationships.

### Limits of mentorship

Despite its benefits, mentorship is not without its limitations, and lack of motivation and poor collaboration at the mentee level have been identified as main contributors to mentorship failure.^28,29^ At the mentor level, lapses in professionalism, failure to acknowledge mentee contributions, and limited experience with mentoring have been cited as threats to mentorship effectiveness.^29^ Beyond these factors, ethical challenges such as power differentials and cultural differences may further complicate mentorship dynamics, particularly in academic and professional settings.^29^

## Conclusion

Recent changes to medical education and changes in the residency application process have fundamentally reshaped the process of matching into dermatology. As objective measures of evaluating candidates have decreased and the application review process has become more subjective in nature, mentorship has moved from a helpful supplement to a vital conduit for success. Now more than ever, who authors an applicant’s letters of recommendation, whether they complete a research year, how preference signals are used, and whether they receive advocacy and sponsorship through mentorship can meaningfully influence an applicant’s chance of success.^30^

Anecdotally, mentors are also now being approached by a substantially greater number of mentees than in previous years, when the ratio of mentees to mentors was more often 1:1 or 2:1.^31^ This demand, which has increased as the specialty of dermatology has risen in popularity, has the potential to lead to burnout among mentors and limit opportunities for individualized guidance.^30,32^ This may disproportionately affect women and underrepresented groups, who may depend more heavily on mentorship to help make career decisions and overcome structural barriers that already make the specialty more difficult to navigate than others.^6,19,33,34^ While it is unrealistic to predict what changes might be on the horizon for the application process, medical training, or dermatology in general, evolution is expected. Thus, mentorship models that are transparent, equitable, and adaptable to these inevitable changes will remain critical regardless of how the landscape continues to shift.

## Conflicts of interest

The authors made the following disclosures: Dr. S.C.T. has served as a consultant, advisory board member and/or speaker for AbbVie, Arcutis, Armis Scientific, Avita, Beiersdorf, Biorez, Bristol-Myers Squibb, Cara Therapeutics, Dior, Eli Lilly, EPI Health, Evolus, Galderma, GloGetter, Hugel America, Incyte, Johnson & Johnson, L’Oreal USA, MedScape, MJH LifeSciences, Pfizer, Piction Health, Sanofi, Scientis US, UCB and Vichy Laboratories. Dr. N.E. has served as a consultant, advisory board member, and/or speaker for Avita, Incyte, VisualDx, Beiersdorf, Unilever, Eli Lilly, Galderma, Pfizer, La Roche-Posay, L’Oreal, McGraw Hill, Dior, Medscape, AbbVie, Takeda, Sanofi, Janssen, Kenvue, and Kao. She has received royalties from McGraw Hill. She has stock options in VisualDx. Dr. Elbuluk has served as a consultant, advisory board member, and/or speaker for Avita, Incyte, VisualDx, Beiersdorf, Unilever, Eli Lilly, Galderma, Pfizer, La Roche-Posay, L’Oreal, McGraw Hill, Dior, Medscape, AbbVie, Takeda, Sanofi, Janssen, Kenvue, Kao. She has received royalties from McGraw Hill. Dr. T.O. has served as a consultant, advisory board member, and/or speaker for Beiersdorf, MJH LifeSciences, Dermatology Times, and Health Central. All other authors have no disclosures.

## Funding

None.

## Author contributions

All authors contributed to the design, conduct, and writing of this review. Each author read and approved the final draft.

## References

[1] Association of American Medical Colleges. New AAMC data on medical school applicants and enrollment in 2024. AAMC; 2025. Available from: https://www.aamc.org/news/press-releases/new-aamc-data-medical-school-applicants-and-enrollment-2024

[2] Grant-Kels JM. Thoughts on dermatology residents who are new parents. Int J Womens Dermatol 2020;6:344–5. 10.1016/j.ijwd.2020.05.006 PMC7224652 32420445

[3] Donovan JC. A survey of dermatology residency program directors’ views on mentorship. Dermatol Online J 2009;15:1. 19930988

[4] Lin G Murase JE Murrell DF Da Cunha Godoy L Grant-Kels JM. The impact of gender in mentor–mentee success: results from the Women’s Dermatologic Society Mentorship Survey. Int J Womens Dermatol 2021;7:398–402. 10.1016/j.ijwd.2021.04.010 PMC8484982 34621951

[5] Farah RS Goldfarb N Tomczik J Karels S Hordinsky MK. Making the most of your mentorship: viewpoints from a mentor and mentee. Int J Womens Dermatol 2020;6:63–7. 10.1016/j.ijwd.2019.12.002 PMC6997903 32042887

[6] Donovan JC. Mentorship in dermatology residency training programs: charting the right course. Dermatol Online J 2009;15:3. 19624981

[7] Wu J Olagunju AT. Mentorship in medical education: reflections on the importance of both unofficial and official mentorship programs. BMC Med Educ 2024;24:1233. 10.1186/s12909-024-06248-7 PMC11523898 39472896

[8] Okoye GA. Supporting underrepresented minority women in academic dermatology. Int J Womens Dermatol 2020;6:57–60. 10.1016/j.ijwd.2019.09.009 PMC6997820 32025561

[9] Nambudiri VE Shi CR Vleugels RA Olbricht SM. Academic dermatology leadership in the United States – addressing the gender gap. Int J Womens Dermatol 2018;4:236–7. 10.1016/j.ijwd.2018.05.003 PMC6322155 30627624

[10] Gold S Blalock TW. Stepping back: impact of pass-fail step 1 scoring on dermatology applicants. Clin Dermatol 2022;40:602–3. 10.1016/j.clindermatol.2022.08.004 35961480

[11] Kovacs LD Zhou AE Korytnikova E. Demystifying the dermatology residency application process, part one-building the application: competition, academic metrics, and extracurricular activities. Clin Dermatol 2026;44:150–6. 10.1016/j.clindermatol.2025.06.003 40592363

[12] Wang A Karunungan KL Story JD Ha EL Braddock CH 3rd. Residency Program Director Perspectives on changes to US Medical Licensing Examination. JAMA Netw Open 2021;4:e2129557. 10.1001/jamanetworkopen.2021.29557 PMC8520127 34652450

[13] Ravi S Kovacs LD Korytnikova E. Demystifying the dermatology residency application process, part two-application season: strategic application, interviewing, and mentorship. Clin Dermatol 2025;44:S0738-081X(25)00185-3. 10.1016/j.clindermatol.2025.06.004 40614906

[14] Costello CM Harvey JA Besch-Stokes JG. The role research gap years play in a successful dermatology match. Int J Dermatol 2022;61:226–30. 10.1111/ijd.15964 34719024

[15] Lipner S Falotico J. Post-residency training instead of medical student research fellowships will improve dermatology patient care and excellence. J Drugs Dermatol 2022;21:917–8. 10.36849/JDD.6898 35946965

[16] Jung J Stoff BK Orenstein LAV. Unpaid research fellowships among dermatology residency applicants. J Am Acad Dermatol 2022;87:1230–1. 10.1016/j.jaad.2021.12.027 34942299

[17] Vasquez R Jeong H Florez-Pollack S Rubinos LH Lee SC Pandya AG. What are the barriers faced by under-represented minorities applying to dermatology? A qualitative cross-sectional study of applicants applying to a large dermatology residency program. J Am Acad Dermatol 2020;83:1770–3. 10.1016/j.jaad.2020.03.067 32244012

[18] McRae C Schroeder A Anderson M Turner L Kole L. How increasing research demands threaten equity in dermatology residency selection and strategies for reform. Cutis 2025;116:82–6. 10.12788/cutis.1265 41183477

[19] Soliman YS Rzepecki AK Guzman AK. Understanding perceived barriers of minority medical students pursuing a career in dermatology. JAMA Dermatol 2019;155:252–4. 10.1001/jamadermatol.2018.4813 PMC6439537 30624570

[20] Sarabipour S Hainer SJ Arslan FN. Building and sustaining mentor interactions as a mentee. FEBS J 2022;289:1374–84. 10.1111/febs.15823 PMC8490489 33818917

[21] Frei E Stamm M Buddeberg-Fischer B. Mentoring programs for medical students--a review of the PubMed literature 2000-2008. BMC Med Educ 2010;10:32. 10.1186/1472-6920-10-32 PMC2881011 20433727

[22] Zerzan JT Hess R Schur E Phillips RS Rigotti N. Making the most of mentors: a guide for mentees. Acad Med 2009;84:140–4. 10.1097/ACM.0b013e3181906e8f 19116494

[23] Manuel SP Poorsattar SP. Mentoring up: twelve tips for successfully employing a mentee-driven approach to mentoring relationships. Med Teach 2021;43:384–7. 10.1080/0142159X.2020.1795098 32715860

[24] Ramani S Kusurkar RA Lyon-Maris J. Mentorship in health professions education – an AMEE guide for mentors and mentees: AMEE Guide No. 167. Med Teach 2024;46:999–1011. 10.1080/0142159X.2023.2273217 37909275

[25] Tuma TT Dolan EL. What makes a good match? Predictors of quality mentorship among doctoral students. CBE Life Sci Educ 2024;23:ar20. 10.1187/cbe.23-05-0070 PMC11235109 38640406

[26] Kim CC Kim EJ Curiel-Lewandrowski C Marks V Maloney M Frieden IJ. A model in dermatology for long-distance mentoring. J Am Acad Dermatol 2013;68:860–2. 10.1016/j.jaad.2012.10.049 23267719

[27] Junn JC Whitman GJ Wasnik AP. Virtual mentoring: a guide to navigating a new age in mentorship. Acad Radiol 2023;30:749–54. 10.1016/j.acra.2022.08.014 36089477

[28] Straus SE Johnson MO Marquez C Feldman MD. Characteristics of successful and failed mentoring relationships: a qualitative study across two academic health centers. Acad Med 2013;88:82–9. 10.1097/ACM.0b013e31827647a0 PMC3665769 23165266

[29] Kow CS Teo YH Teo YN. A systematic scoping review of ethical issues in mentoring in medical schools. BMC Med Educ 2020;20:246. 10.1186/s12909-020-02169-3 PMC7395401 32736552

[30] Wasong SJH Miller JJ Zaenglein AL. Does a predermatology fellowship increase the chance to match in dermatology? J Am Acad Dermatol 2008;59:535–6. 10.1016/j.jaad.2008.05.022 18694691

[31] Hussain K Patel N Fearfield L Roberts N Staughton R. Mentorship in dermatology: a necessity in difficult times. Clin Exp Dermatol 2022;47:622–3. 10.1111/ced.15071 34989010

[32] Wilkinson ST. Is it normal to experience burnout as a mentor? Hosp Pharm 2023;58:5–6. 10.1177/00185787221133802 PMC9837319 36644748

[33] Tavarez MM Baghdassarian A Bailey J. A call to action for standardizing letters of recommendation. J Grad Med Educ 2022;14:642–6. 10.4300/JGME-D-22-00131.1 PMC9765898 36591418

[34] Isaq NA Bowers S Chen ST. Taking a “step” toward diversity in dermatology: de-emphasizing USMLE Step 1 scores in residency applications. Int J Womens Dermatol 2020;6:209–10. 10.1016/j.ijwd.2020.02.008 PMC7330438 32637547

